# A Simple Screen to Identify Promoters Conferring High Levels of Phenotypic Noise

**DOI:** 10.1371/journal.pgen.1000307

**Published:** 2008-12-19

**Authors:** Nikki E. Freed, Olin K. Silander, Bärbel Stecher, Alex Böhm, Wolf-Dietrich Hardt, Martin Ackermann

**Affiliations:** 1 Institute of Integrative Biology, Eidgenössische Technische Hochschule (ETH) Zurich, Zurich, Switzerland; 2 Institute of Microbiology, Eidgenössische Technische Hochschule (ETH) Zurich, Zurich, Switzerland; 3 Biozentrum, University of Basel, Basel, Switzerland; Université Paris Descartes, INSERM U571, France

## Abstract

Genetically identical populations of unicellular organisms often show marked variation in some phenotypic traits. To investigate the molecular causes and possible biological functions of this phenotypic noise, it would be useful to have a method to identify genes whose expression varies stochastically on a certain time scale. Here, we developed such a method and used it for identifying genes with high levels of phenotypic noise in *Salmonella enterica* ssp. I serovar Typhimurium (*S.* Typhimurium). We created a genomic plasmid library fused to a green fluorescent protein (GFP) reporter and subjected replicate populations harboring this library to fluctuating selection for GFP expression using fluorescent-activated cell sorting (FACS). After seven rounds of fluctuating selection, the populations were strongly enriched for promoters that showed a high amount of noise in gene expression. Our results indicate that the activity of some promoters of *S.* Typhimurium varies on such a short time scale that these promoters can absorb rapid fluctuations in the direction of selection, as imposed during our experiment. The genomic fragments that conferred the highest levels of phenotypic variation were promoters controlling the synthesis of flagella, which are associated with virulence and host–pathogen interactions. This confirms earlier reports that phenotypic noise may play a role in pathogenesis and indicates that these promoters have among the highest levels of noise in the *S.* Typhimurium genome. This approach can be applied to many other bacterial and eukaryotic systems as a simple method for identifying genes with noisy expression.

## Author Summary

According to the conventional view, the characteristics of an organism are determined by nature and nurture—by its genes and by the environment it lives in. Consequently, one would expect that two organisms that share the same genes and live in the same environment have identical characteristics. Recently it has become clear that this expectation is often not borne out; clonal families of simple organisms living under constant conditions often show variation in biological traits and sometimes even have markedly different properties and do different things. In order to investigate molecular causes and possible biological functions of such phenotypic noise, it would be very valuable to have a simple and fast method for identifying biological traits that are particularly noisy. Here, we developed such a method, and screened for noisy traits in the bacterial pathogen *Salmonella typhimurium* that vary at a time scale of one day. We found that traits involved in interaction with the host are particularly noisy, suggesting that phenotypic noise might be important in pathogenesis. This method can be readily adopted for other organisms and might contribute to elucidating the role of noise in biology.

## Introduction

Clonal populations of unicellular organisms growing under constant conditions often show substantial variation in phenotypic traits. The rate at which some of these traits vary is so high that it cannot result from mutational change. Rather, this phenotypic noise has been shown to result from chance events in the cells, namely random fluctuation in the transcription and translation of genes [1]–[3]. Most of the research on phenotypic noise focuses on two questions. First, what are the molecular processes underlying this phenomenon? Second, are there cases in which phenotypic noise is beneficial? Can it provide a genotype with new biological functions and improve the chance that it will survive and reproduce?

To further our understanding of the biological significance of phenotypic noise, it would be helpful to have a simple method to identify genes whose expression varies stochastically at a given timescale and under specific environmental conditions. So far, most of the research on phenotypic noise was based on the detailed analysis of individual biological traits [4]–[6]. It is interesting to complement these studies with a global analysis, so that one can ask whether the traits studied so far are indeed particularly noisy, or whether a substantial fraction of all genes show such high levels of noise. One possibility for a global analysis of phenotypic noise is the exhaustive characterization of ordered libraries of strains marked with reporter proteins [7]. Here, we have established a simple alternative that allows identifying promoters whose activity varies on a specific time-scale; we used this method to identify promoters in the bacterial pathogen *S.* Typhimurium that switch between active and inactive over the course of a few generations.

The method is based on subjecting a promoter library to selection for high levels of random variation on a short time scale. The screen was initiated with a genomic library consisting of short genomic fragments upstream of a gene encoding green fluorescent protein (GFP). Cells carrying a fragment with an active promoter expressed GFP. In order to select for promoters with a high level of phenotypic noise, we used fluorescence-activated cell sorting to select cells based on the cellular concentration of GFP, and alternated between selecting for high levels of GFP, and selecting for low levels of GFP. There was no signal indicating the direction of selection during a given round of the selection experiment; one would thus expect that promoters that randomly switch between expressing and not expressing GFP would increase in frequency.

This screen led to a strong enrichment of promoters with high levels of noise. The promoters that showed the highest levels of noise were found to be flagellar promoters, which are involved in the interaction with the host. These promoters have previously been reported to be heterogeneously expressed in clonal populations of *S*. Typhimurium. Our screen demonstrates that these promoters stand out in terms of the level of noise, and that they vary on a very short timescale. This method thus offers a simple and powerful approach to identify genes with high levels of noise, and allows for easily modulating timescale and environmental conditions under which such phenotypic noise manifests.

## Results/Discussion

We established a population of approximately 7×10^6^ *S.* Typhimurium clones containing a library of genomic fragments ranging in size from 400 bp to 1200 bp linked to a GFP reporter (see Methods). In order to enrich for clones exhibiting increased levels of phenotypic noise in GFP concentration, we used a regime of alternating selection. Cells were grown into exponential phase, and subjected to selection on GFP concentration in a fluorescence-activated cell sorter (FACS). First, we selected only those clones having a level of GFP expression in the highest 5% of the population; these clones were saved and used to inoculate fresh cultures that were grown overnight. In the next step, the opposite selection regime was imposed, such that only those clones having a level of GFP expression in the lowest 5% of the population were saved and grown. It is also possible to first select cells expressing low levels of GFP, and then high, which hypothetically would result in noisy promoters with lower average expression.

This process of fluctuating selection was repeated, until a total of seven alternating selection events had occurred. The fluctuating selection regime was performed on five separate populations; five control populations were also exposed to the same regime of growth and FACS sorting, but no selection occurred for the level of GFP concentration (a random subset of cells covering the entire range of GFP fluorescence was saved and grown). After the seven rounds of selection, clones from all populations were plated onto agar plates. Twenty-four clones from each of the ten populations were randomly selected for future analyses.

### Phenotypic Noise Is a Stable and Consistent Property of a Clone

Selection for increased phenotypic noise can only be successful if the level of variation is a stable property of a clone. We thus first asked whether the level of phenotypic noise in GFP expression was a stable and consistent trait in these clonal isolates. We used the 240 frozen clonal stocks described above to seed fresh cultures of cells, and analyzed GFP concentration for about 5×10^5^ cells per clone (see Methods). We repeated the same procedure on a different day, and also gathered data on GFP expression for the same set of 240 clones. Phenotypic noise was quantified using the coefficient of variation in GFP expression from a subset of cells similar in size, shape, and cellular complexity (see Methods). We found that the level of phenotypic variation observed for a given clone on day 1 was highly correlated with the level observed on day 2 (r^2^ = 0.748, p<0.001). This shows that the level of phenotypic noise is a consistent property of a clone (presumably reflecting the noise of the promoter on the genomic fragment it contains), and that this property is stably maintained in clonal populations that are repeatedly grown from an individual cell.

### Fluctuating Selection Enriches for Clones Exhibiting Increased Phenotypic Noise

**Figure 1:**
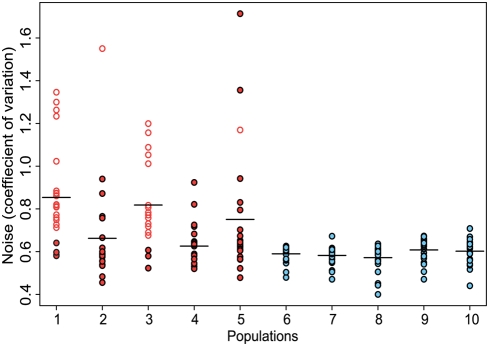
Noise in GFP expression in clones from selected and control populations. Clones from selected populations (red) show a higher level of noise than do clones from control populations (blue) (univariate GLM, p = 0.016). Open circles indicate clones that contain the promoter sequence for the *fliC* gene driving GFP expression, which were significantly enriched in the selected populations. Each data point represents the coefficient of variation of the GFP expression of several thousand individual cells.

**Figure 2:**
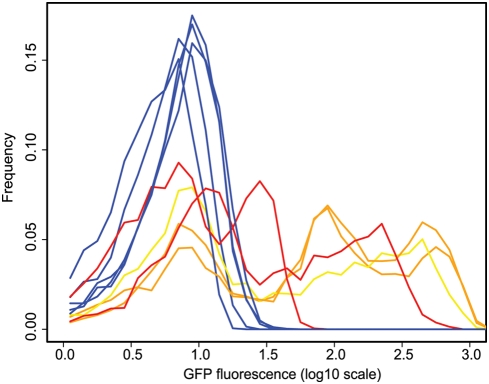
Histograms of GFP expression from clones exhibiting the highest level of noise in each population. Clones from each of the ten populations were ranked according to the amount of noise in GFP expression produced. A histogram of GFP expression was plotted for a single clone from each population with the highest level of noise. Clones from selected populations (red, orange, and yellow lines) show a much higher level of noise than the control those from control populations (blue lines). Clones containing the *fliC* promoter are orange and a clone containing the *flgK* promoter is yellow.

Next, we asked whether fluctuating selection had led to an enrichment of clones exhibiting larger amounts of stochastic phenotypic variation. We compared the clones from the five selected populations to the clones from the five control populations. Among the clones from the selected populations, a sizable fraction showed high coefficients of variation in fluorescence, which is a measure of stochastic phenotypic variation. In contrast, the control population did not contain any clones with high coefficients of variation (Figure 1, Figure 2). An analysis of variance showed that the average coefficient of variation was higher in the selected than in the control populations (p-value = 0.016, by GLM univariate). This demonstrated that fluctuating selection on fluorescence enriched for strains with high levels of stochastic variation in this trait.

### Promoters of Genes Involved Flagellar Synthesis Exhibit High Levels of Phenotypic Noise

This simple selection scheme is thus a good tool for enriching for noisy promoters. Identifying the genes controlled by these promoters then gives a fairly unbiased look at genes whose expression is particularly variable, and might thereby provide new insights into the biological role of noise. In order to identify these genes, we sequenced the library inserts from the 240 frozen clonal stocks (24 from each experimental population). We found that the clones exhibiting the highest levels of variation were dominated by two promoter sequences that regulate genes involved in flagellar synthesis, namely *fliC* and to a lesser extent *flgK* (Figure 1; Table S1). On the other hand, none of the inserts sequenced from the control populations contained promoters associated with the expression of flagellar or related genes, suggesting that this result was not due simply to overrepresentation of flagellar promoters in the genomic library.

We focused on *fliC* for two tests of the robustness of our results. First, we tested whether the *fliC* promoter is also noisy in the native chromosomal context. To do so, we constructed a transcriptional fusion of *gfp* to the *fliC* promoter at its native location in the chromosome. Clones from this chromosomal construction showed very similar levels of phenotypic noise to the plasmid-based *fliC* promoter (Figure S1, Text S1). Second, we asked whether GFP expression from the plasmid is correlated with actual protein production of *FliC*. Clones containing the *pfliC-GFP* insert in the plasmid pM968 with high levels of variation in GFP expression were sorted into three fractions (expression of GFP, no expression of GFP, and cells expressing all levels of GFP). Western blot analysis with *anti-FliC, anti-fljK* antibodies on these three cell fractions confirmed that GFP expression is positively correlated with FliC protein production. (Figure S2 and Text S1). These two experiments indicate that the levels of noise we measured are, at least in the case of *fliC*, not an artifact of the plasmid-based reporter system, but do reflect actual differences in protein production between cells.

### High Levels of Phenotypic Variation in the *fliC* Promoter Are Not Due To Genetic Phase Variation

**Figure 3:**
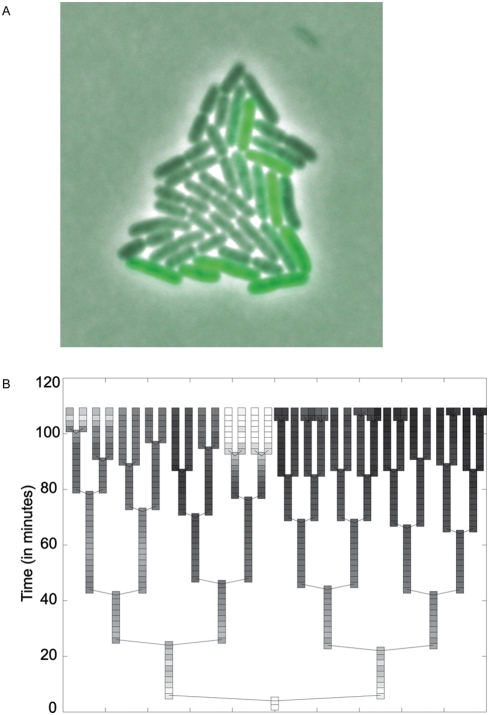
Phenotypic noise in a microcolony in *gfp* expression from the *fliC* promoter. A. An image of a microcolony containing the plasmid-borne *fliC* promoter driving expression of GFP. The colony was started from a single cell and grown for about 6 generations. B. A lineage tree of this microcolony with GFP expression plotted in green (light colored boxes represent high levels of GFP, and dark boxes represent low levels), illustrating the temporal pattern of switching of the *fliC* promoter. The image and the lineage tree are based on Movie S1.

The variation in the expression of GFP under the control of flagellar promoters observed here is reminiscent of a genetic switch known as phase variation. *S.* Typhimurium express two distinct flagellin proteins, FliC and FljB [8], and switches between the two flagellar types using a site-specific recombination event in the chromosome. Can phase variation account for the phenotypic noise that we measured in the clones harboring the flagellar promoters? Site-specific recombination occurs at a rate of 10^−3^ to 10^−5^ per cell division [9]–[11]. In a clonal population grown from a cell in one phase, it thus takes many divisions until recombination-mediated phase variation has a reasonable likelihood of occurring. However, this is not what we observed in the clones with flagellar promoters: populations grown from single cells quickly attained substantial proportions of cells with both high and low expression of GFP (Figure 3, Movie S1). In contrast, clones isolated from the control populations maintained similar levels of GFP expression (Figure S3, Movie S2). This suggests that it is unlikely that the variation observed in these clones can be attributed to phase variation.

As a direct test of the effect of phase variation on stochastic phenotypic variation, we transformed the plasmid with the *fliC* promoter controlling GFP expression into a host strain that is incapable of phase variation [8] and into a wildtype strain. The resulting populations still showed strong variation in the amount of GFP between cells, and the coefficient of variation was not significantly different between the plasmid containing the *fliC* promoter in the wildtype background and the strain incapable of phase variation (t-test, p = 0.199, 95% Mean CV for wildtype background is 1.07, mean CV for *fljAB* promoter “locked” off background is 0.95, 95% Confidence interval for the difference is 0.068 and −0.299). This demonstrates that phase variation is not the main reason for the phenotypic noise observed here, and is most likely not involved.

### Possible Biological Roles of Phenotypic Noise in *S*. Typhimurium

Having identified promoters that are particularly variable, one can then ask whether variability in these promoters might serve a biological function. This question can be addressed by functional studies of the genes whose expression is particularly noisy. However, first insights can be gained from looking at the types of promoters that showed the highest levels of stochastic phenotypic variation.

By far the highest level of phenotypic noise observed in our experiment comes from flagellar promoters, most notably, *fliC*. This supports a previous report that the expression of *FliC* is heterogeneous in clonal populations of *S.* Typhimurium [12]. Bacterial flagella are required for colonization and tissue invasion [13],[14] and they interact with the host immune system in a myriad of ways, eliciting both innate and specific immune responses [15]–[18]. That variation in the expression of flagella might be advantageous is a well-established concept [19]; it usually refers to variation mediated by a site-specific recombination event, but has recently also been extended to variation that presumably does not involve changes in the DNA sequence [12],[20].

The advantage that is usually postulated is mediation between conflicting selection pressures on flagellar expression in the host. During initial stages of gut infection by *S.* Typhimurium, flagella are instrumental for swimming towards the host's epithelial mucus layer [14]. During later stages of infection, a switch towards not expressing flagellin might be of advantage for bacteria that have invaded epithelial tissue, as it avoids recognition by the innate immune system [TLR5, Naip/Nalp][21]. There is a second possible biological function of phenotypic noise in flagella and other factors involved in the interaction with the host. A recent study suggested that heterogeneous expression of these traits in clonal populations of *S.* Typhimurium promotes the division of labor between two phenotypically different subpopulations. One subpopulation invades the gut tissue and elicits an inflammation of the gut; the other subpopulation remains in the gut and benefits from the fact that the inflammation reduces competition from commensal bacteria [22].

Two main insights emerge from this study. The first insight is that the activity of some *S.* Typhimurium promoters varies on such a short time scale that these promoters can absorb rapid fluctuations in the direction of selection, as imposed during our experiment. This is an important experimental test of one of the main ideas for why phenotypic noise can be adaptive: variation in the phenotypes encoded by a single genotype can increase the long-term growth rate of this genotype in fluctuating environments [23],[24].

The second insight is methodological: fluctuating selection is a simple and fast tool to screen large pools of individuals in order to identify variable promoters in unicellular organisms, and thus complements exhaustive characterizations of individual genes [7]. Exhaustive characterizations require the construction of ordered libraries in which fluorescent markers are transcriptionally or translationally fused to every gene, as well as individual measurement of all resulting strains. In contrast, the method presented here only requires the relatively simple construction of a random genomic library, and sorting of the pooled library. It is thus also applicable to eukaryotic systems and organisms that are not genetic model systems, as long as they can be stably transformed. It should thus be feasible to identify noisy promoters in a diverse range of environmental, commensal, and pathogenic organisms, and to ask whether differences in the lifestyle lead to consistent differences in the types of genes that are variable.

One particular advantage of this tool is that the time-scale at which the direction of selection changes can be varied. By changing the direction of selection every few cell divisions, on can impose selection for promoters that switch at a very high rate; changing the direction of selection less frequently selects for promoters that switch at lower rates. It should thus be possible to identify promoters that vary at different time scales, and to investigate whether they might be associated with responses to environmental conditions that vary at different frequencies.

Once noisy promoters are identified, functional studies are needed to investigate the biological consequences of their variation. This might lead toward new answers to one of the fundamental and most challenging questions about the biology of noise – whether phenotypic noise is beneficial, and what its possible benefits might be.

## Materials and Methods

### Growth of Strains

Strains were grown at 37°C on LB agar plates or in 1 ml of liquid LB broth in 5ml polystyrene round bottom tubes (BD Falcon), with shaking at 200 rpm until mid-exponential phase. Ampicillin (Sigma) was used at a concentration of 100 µg/ml in strains containing plasmid pM968 or its derivatives.

### Construction of the Plasmid Library

A plasmid library (7×10^6^ clones) was constructed by partially digesting *S.* Typhimurium SL1344 wildtype [25] chromosomal DNA with *Bsp*143I. Fragments within a size range of 400 bp to 1200 bp were ligated into *Bam*HI digested pM968. This plasmid is low copy number promoter-less derivative of pBAD24 containing promoterless *gfpmut2,* described in [26]. Plasmids were transformed into *E. coli* Χ6060, re-isolated by standard methods and electrotransformed into *S.* Typhimurium M324 (Δ *aroA invC*::*aphT ssaV*::*cat* [26]). Colonies were selected by growth on LB agar plates containing Ampicillin, harvested, and pooled.

### Growth for Flow Cytometry and Cell Sorting

A 1∶1000 dilution of an overnight culture of the plasmid library was split into ten equal populations; five populations were assigned to “selected” and five to “control” groups. Cells were grown for 2 hours to reach exponential growth. Cultures were spun down at 3000× g for five minutes at 4°C. Growth media was removed and cultures were re-suspended in ice cold PBS. Cells were kept on ice until sorted or analyzed as described below.

### Fluctuating Selection using Cell Sorting

We subjected the plasmid library to fluctuating selection on fluorescence intensity, where selection for bright cells alternated with selection for dim cells.

Cells were sorted using fluorescence-activated cell sorting (FACS) with FACS–Diva sorting software (Becton Dickinson, CA). Immediately prior to sorting, 5×10^5^ cells from each of the ten populations were analyzed for GFP expression. Based on this analysis, on the first day, a gate was drawn for each population to include either the highest 5% of cells expressing GFP, or a gate that covered the entire range of GFP expression, for selected and control lines, respectively. From each gated area, 1×10^5^ cells were collected into a sterile well of a 24-well plate. Cells were collected at a 2.0 flow rate and sorted on the basis of “single cell” and “purity”. After sorting, cells were spun at 3000* g* for ten minutes and any FACS buffer was removed. Cells were re-suspended in 1ml LB media containing Ampicillin and grown overnight. The following day the process was repeated; however the gates for the selected populations included only the lowest 5% of cells expressing GFP. This process was repeated for a total of seven rounds of selection, with gates being drawn for selected populations in a fluctuating manner: selection on the highest 5% of GFP expression, then lowest 5%, and back again to the highest 5% of the total. After the 5^th^ round of selection all populations were placed at 4°C for 48 hours. After this time, selection was resumed as normal. After all rounds of selection were completed, the populations were plated on LB agar plates containing Ampicillin, and 24 single colonies from each experimental population were randomly selected (240 clones in total). These were grown overnight in 1ml of LB containing Ampicillin and frozen at −80°C in 15% glycerol.

### Analysis and Data Processing

One day prior to analysis, the 240 frozen clonal stocks were used to inoculate 1ml of medium in 5ml polystyrene round bottom tubes (BD Falcon) and prepared in the same manner as described above (Growth for cytometry and cell sorting). For each clone, 5×10^4^ cells were analyzed for GFP expression on the FACS Calibur (BD, CA).

Raw data was exported from FlowJo 4.6.1 software (TreeStar, Ashland, OR) into custom software. The software was used to exclude data deemed to be extraneous and for performing calculations relating to noise in fluorescence intensity.

The following conventions were applied to calculate variation in GFP expression and to limit the influence from cellular aggregates, cell detritus, and undefined values. Modified from Newman et al [7]:

1. All SSC, FSC, and fluorescence zero values were excluded.
2. Data was excluded that fell within the forward scatter (FSC) and side scatter (SSC) region where significant counts appeared in “buffer only” controls.
3. Extreme values of FSC and SSC were excluded (the highest and lowest 2.5% of events) from total counts in order to limit influence from cell detritus and cell aggregates.
4. FSC and SSC medians were calculated and a series of circular gates expanding out from the FSC and SSC medians were applied. For each gate size the coefficient of variation (CV) was calculated for fluorescence. A single gate size was then chosen for all analyses; this gate resulted in the lowest average CV (in order to maintain a conservative estimate of noise) yet contained enough cells for robust analysis (a minimum of 950 cells).
5. Extreme values of the fluorescence channel (FL1) (the highest 1.0% of events) were excluded to limit only a very small number of cells having undue effects on the values of the mean and CV.

When calculating the correlation between the coefficients of variation in fluorescence on two consecutive days, two data points were excluded from the analyses because they were more than 3 standard deviations away from expected values.

### Sequencing

The following primers (F: 5′ GTCAGAGGTTTTCACCGTCATCAC 3′. R: 5′CAAGAATTGGGACAACTCCAGTG 3′) were used to PCR amplify the genomic segments inserted into plasmid pM968. Both primers anneal to regions on pM968 that flank the insert region. Inserts were sequenced using the reverse primer. The insert sequences were blasted against the genomic sequence of *Salmonella typhimurium* LT2 genomic and plasmid sequence (accession numbers NC_003197 and NC_003277), and the single best hit was retained as a hypothetical promoter. For each of these hypothetical promoters, the two nearest downstream genes were checked to see if either were oriented in the same direction as the hypothetical promoter. If either of these genes were oriented in the correct direction, the name and distance to the closest gene was noted. If neither of these genes were oriented in the correct direction, we concluded that it was unlikely that the insert sequence was actively driving transcription.

### Cell Tracking and Analysis of GFP Expression in Microcolony Formation

Cell tracking software was used to track cell lineages and analyze GFP expression in individual cells during microcolony growth as described in [27].

## Supporting Information

Figure S1 Comparison of noise in expression of chromosomal-based and plasmid-based *fliC* promoter. A. Comparison of noise, as given by coefficient of variation in GFP expression, from the *fliC* promoter on the plasmid pM968 and in the native location on the chromosome of strain M557. Strain M557 (containing no *gfp* gene) and a *rpsM* promoter fused to *gfp+* [27] inserted in the chromosome of strain M557 serve as controls. There is no significant difference in noise between plasmid-based and chromosome-based expression of GFP under the control of the fliC promoter. B. Histograms of GFP expression from the *fliC* promoter on the plasmid pM968 (blue lines) and in the native location on the chromosome (green lines). These two strains differ in the average expression level and in the pattern of distribution of the expression levels in the population. Strain M557 containing no *gfp* gene (black line) and a *rpsM* promoter fused to *gfp+* (red line) inserted in the chromosome of strain M557 serve as controls.
(0.61 MB TIF)

Figure S2 Western blot analysis shows that GFP expression correlates with the expression of *FliC*. Cells containing the *pfliC::gfp* construct in plasmid pM968 were sorted based on expression of GFP using the FACS. Cells were sorted into three fractions, each containing the same number of cells: The first fraction contained cells with high levels of fluorescence; the second fraction contained cells whose fluorescence did not exceed background; the third fraction was a random sample of cells, chosen irrespective of their level of fluorescence. Cells were subjected to western blot analysis with staining using *anti-FliC*, -*FljB* antibodies and reprobed with *anti-OmpC* as a loading control. Only cells with high levels of GFP expression of GFP showed a band when stained with *anti-FliC*, indicating that GFP expression positively correlates with production of *FliC* protein. It is unclear why the fraction containing all cells does not also show a band; however, the lower intensity of the *anti-OmpC* band of this fraction and the fact that this fraction contains many cells that do not express gfp suggests that the *anti-FliC* band might be too faint to see.
(1.34 MB TIF)

Figure S3 Lineage tree of microcolony growth and expression pattern of the *dcm* promoter. GFP expression is plotted in grey (light colored boxes represent high levels of GFP, and dark boxes represent low levels), illustrating the temporal pattern of switching of the *dcm* promoter, isolated from a control population. The image and the lineage tree are based on Movie S2.
(0.56 MB TIF)

Table S1 Sequenced inserts from selected and control populations. Sequence data from the 240 clones used for analysis.
(0.07 MB XLS)

Text S1 Supporting information containing supplementary materials and methods as well as supplementary figure legends.
(0.06 MB DOC)

Movie S1 Time-lapse movie showing GFP expression under the control of the *fliC* promoter during the growth of a microcolony. GFP is under the control of *fliC* promoter on plasmid M956. This movie lasts for 106 minutes in real time. The phase and fluorescent images have been merged; a lineage reconstruction of this movie can be seen in Figure 3 in the main text.
(0.39 MB MOV)

Movie S2 Time-lapse movie showing GFP expression under the control of the *dcm* promoter during the growth of a microcolony. GFP is under the control of the *dcm* (a DNA cytosine methylase) promoter on plasmid M956. This clone was isolated from a control population. This movie lasts for 178 minutes in real time. The phase and fluorescent images have been merged; a lineage reconstruction of this movie can be seen in Figure S3.
(1.37 MB MOV)
