# Supplementary figures and images for: A Simple Screen to Identify Promoters Conferring High Levels of Phenotypic Noise
Source: PLoS Genet. 2008 Dec 19;4(12):e1000307. doi: 10.1371/journal.pgen.1000307 (PMC2588653; doi:10.1371/journal.pgen.1000307)

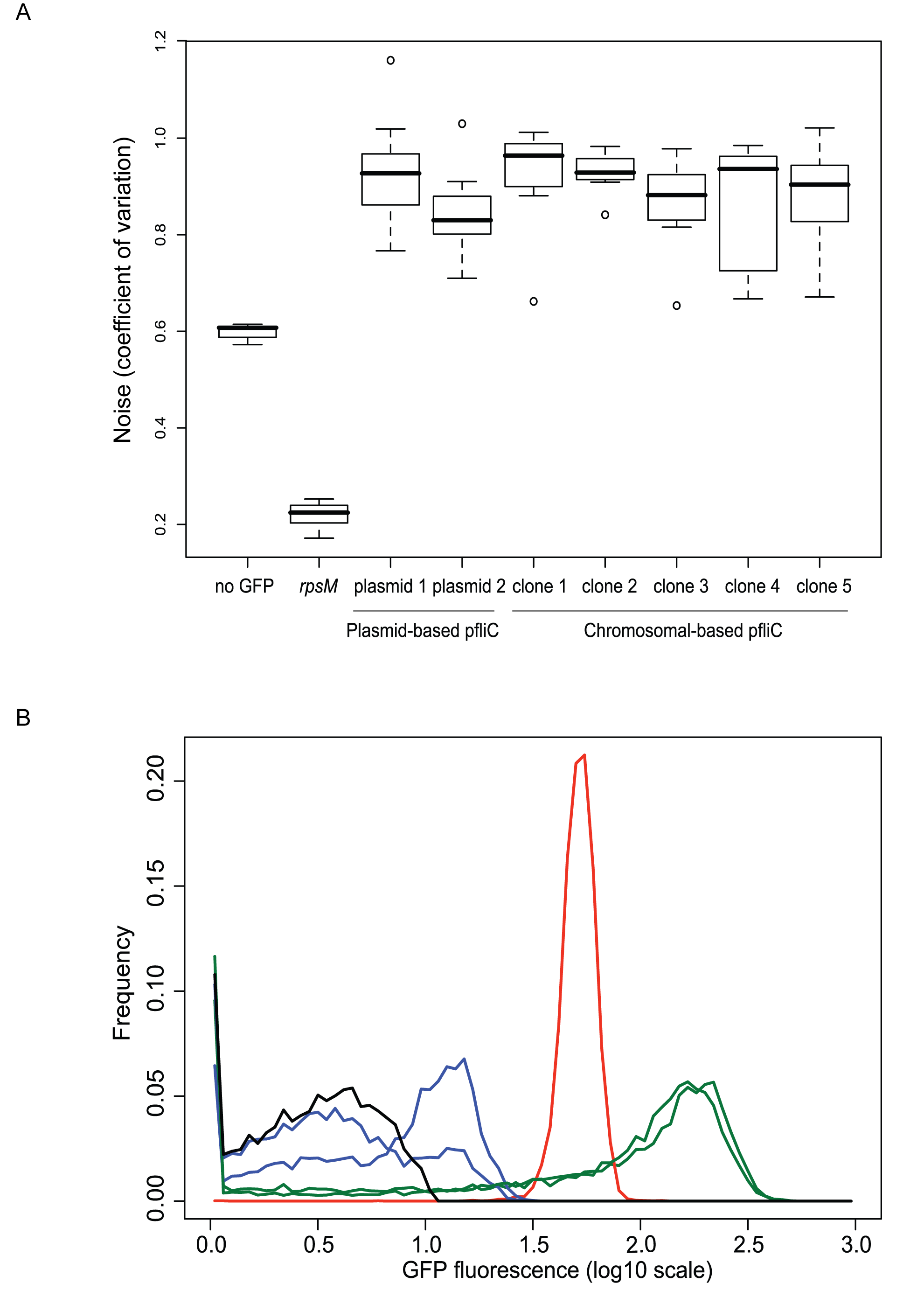

Supplement: Figure S1 — Comparison of noise in expression of chromosomal-based and plasmid-based fliC promoter. A. Comparison of noise, as given by coefficient of variation in GFP expression, from the fliC promoter on the plasmid pM968 and in the native location on the chromosome of strain M557. Strain M557 (containing no gfp gene) and a rpsM promoter fused to gfp+ [27] inserted in the chromosome of strain M557 serve as controls. There is no significant difference in noise between plasmid-based and chromosome-based expression of GFP under the control of the fliC promoter. B. Histograms of GFP expression from the fliC promoter on the plasmid pM968 (blue lines) and in the native location on the chromosome (green lines). These two strains differ in the average expression level and in the pattern of distribution of the expression levels in the population. Strain M557 containing no gfp gene (black line) and a rpsM promoter fused to gfp+ (red line) inserted in the chromosome of strain M557 serve as controls. (0.61 MB TIF) [file pgen.1000307.s001.tif]

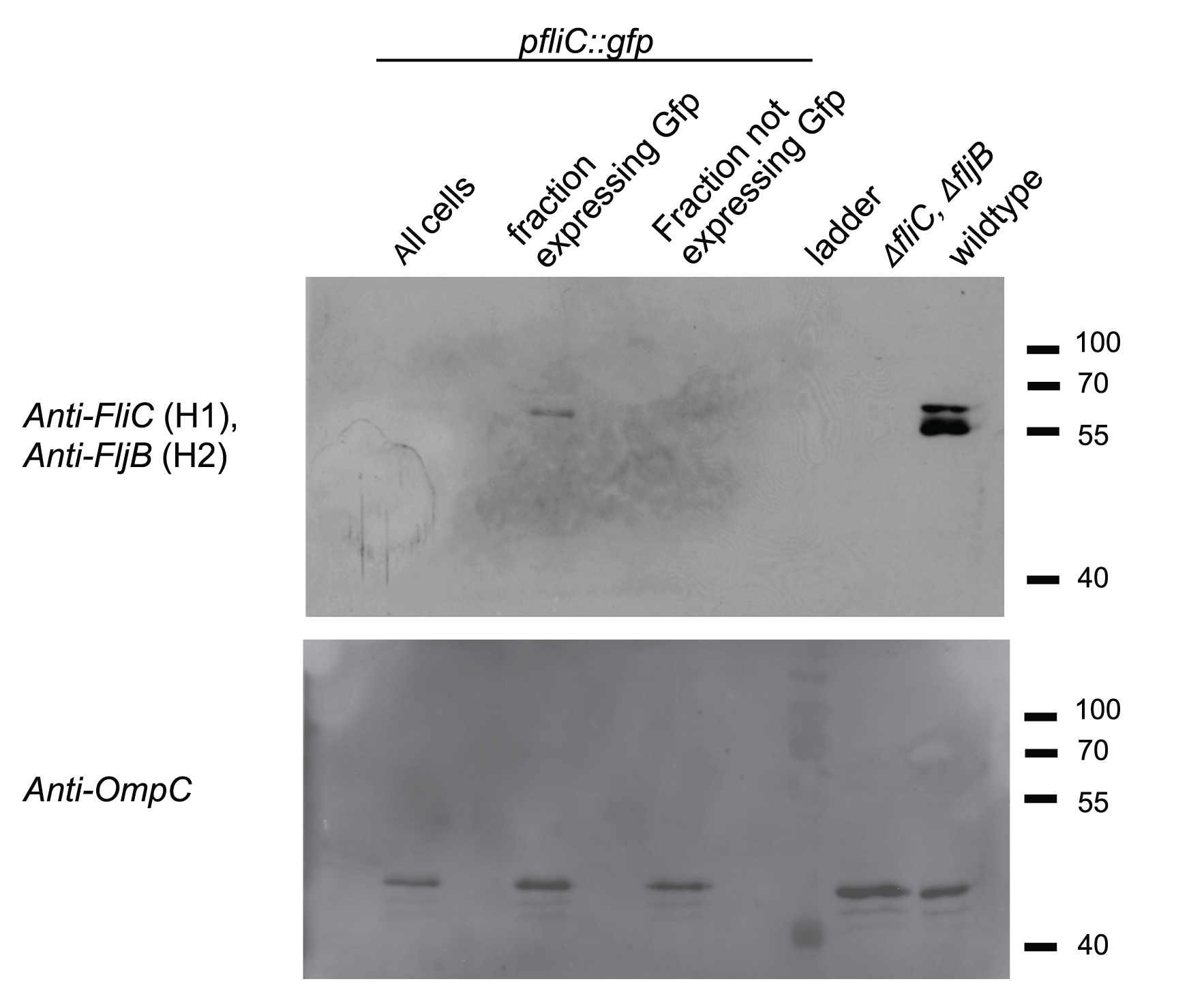

Supplement: Figure S2 — Western blot analysis shows that GFP expression correlates with the expression of FliC. Cells containing the pfliC::gfp construct in plasmid pM968 were sorted based on expression of GFP using the FACS. Cells were sorted into three fractions, each containing the same number of cells: The first fraction contained cells with high levels of fluorescence; the second fraction contained cells whose fluorescence did not exceed background; the third fraction was a random sample of cells, chosen irrespective of their level of fluorescence. Cells were subjected to western blot analysis with staining using anti-FliC, -FljB antibodies and reprobed with anti-OmpC as a loading control. Only cells with high levels of GFP expression of GFP showed a band when stained with anti-FliC, indicating that GFP expression positively correlates with production of FliC protein. It is unclear why the fraction containing all cells does not also show a band; however, the lower intensity of the anti-OmpC band of this fraction and the fact that this fraction contains many cells that do not express gfp suggests that the anti-FliC band might be too faint to see. (1.34 MB TIF) [file pgen.1000307.s002.tif]

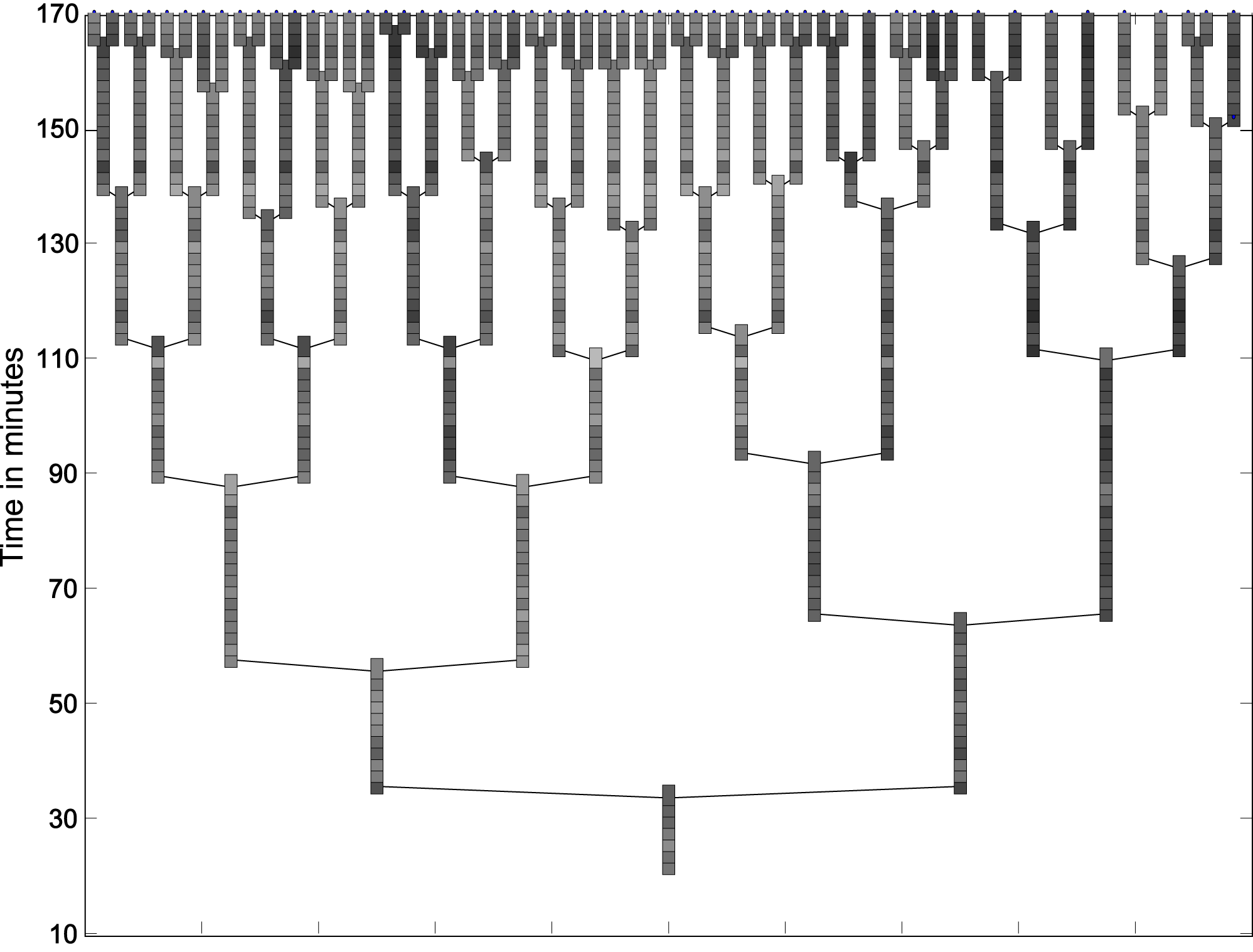

Supplement: Figure S3 — Lineage tree of microcolony growth and expression pattern of the dcm promoter. GFP expression is plotted in grey (light colored boxes represent high levels of GFP, and dark boxes represent low levels), illustrating the temporal pattern of switching of the dcm promoter, isolated from a control population. The image and the lineage tree are based on Movie S2. (0.56 MB TIF) [file pgen.1000307.s003.tif]
