# Supplementary material for: A Simple Screen to Identify Promoters Conferring High Levels of Phenotypic Noise
Source: PLoS Genet. 2008 Dec 19;4(12):e1000307. doi: 10.1371/journal.pgen.1000307 (PMC2588653; doi:10.1371/journal.pgen.1000307)
Supplement: Text S1 — Supporting information containing supplementary materials and methods as well as supplementary figure legends. (0.06 MB DOC) [file pgen.1000307.s005.doc]

**Supplementary Materials and Methods**

*Construction of single copy chromosomal insertion of GFP under control of the* fliC *promoter*

The following primers (F: 5’ GGCAACAGCCCAATAACATCAAGTTGTAATTGATAAGGAAAAGATCATGAGTAAAGGAGAAGAACTTTTC 3’ and R: 5’ CGCTGCCTTGATTGTGTACCACGTGTCGGTGAATCAATCGCCGGATTATTACGCCCCGCCCTGCCA 3’ were used to PCR amplify *gfp*+ and chloramphenicol resistance cassette from strain JH3016 [1]. Amplified DNA was electroporated into strain M557 [2] containing pKD46 for lambda red recombination as described in [3].Constructs were cured of the pKD46 plasmid by growth at 43°C on non-selective plates. Final clones were tested for sensitivity to ampicillin. Constructs were sequenced to confirm proper orientation and insertion at the *fliC* locus.

*Comparison of noise in expression of chromosomal-based and plasmid based fliC promoter*

Two plasmids isolated independently from the plasmid library containing the fliC promoter and five independent chromosomal constructs of *fliC::gfp+* were used for analysis. Plasmids and chromosomal inserts were measured in the background of strain M557. *rpsM-gfp+* [1] in the M557 background was used as a control known to have low levels of noise, and M557 (no GFP) was used as a negative control. All clones were streaked onto agar plates and ten single colonies were randomly chosen. Each colony was grown up and measured using the FACS as described in the Materials and Methods section of the manuscript. Analysis was also done as described in the text. Noise levels, as given by the coefficient of variation in GFP expression, were compared (See Figure S2A). We found that overall GFP expression was lower for chromosomal-based expression as compared to plasmid-based. The noise profiles also showed differences between the two types (see Figure S2B). There was no significant difference in the noise of GFP expression between plasmid-based and chromosomal-based GFP expression under the control of the *fliC* promoter

*Western Blot analysis of cells sorted based on GFP expression*

We performed a western blot analysis using *anti-FliC, -FljB* antibodies and *anti-OmpC* (as a loading control) to test whether GFP expression from the *fliC* promoter on the plasmid (*pfliC::gfp*) does accurately represent the concentration of *FliC* protein in the cell. A clonal population of cells containing the *pfliC::gfp* contruct in plasmid pM9568 exhibiting noisy expression were sorted with the FACS into three fractions, each fraction containing 1.4x 10^7^ cells. The first fraction contained cells with high levels of fluorescence (above the 55^th^ percentile); the second fraction contained cells whose fluorescence did not exceed background (below the 35^th^ percentile); the third fraction was a random sample of cells, chosen irrespective of their level of fluorescence. Cells were concentrated into 15ul and 5ul of Laemmli buffer was added. Cells were heated to 95°C for 5 minutes and then frozen at -20°C. Cell fractions were then subjected to western blotting, with staining for *anti-FliC*, -*FljB*, then reprobed with staining with monoclonal mouse antibody (CM95) *OmpC*-antibody as a control for total number of cells. The western blotting shows that GFP expression does correlate with the expression of *FliC*. This is indicated in Figure S2.

1. Hautefort I, Proenca MJ, Hinton JCD (2003) Single-copy green fluorescent protein gene fusions allow accurate measurement of Salmonella gene expression in vitro and during infection of mammalian cells. Applied and Environmental Microbiology 69: 7480-7491.

2. Hapfelmeier S, Stecher B, Barthel M, Kremer M, Muller AJ, et al. (2005) The Salmonella Pathogenicity Island (SPI)-2 and SPI-1 Type III Secretion Systems Allow Salmonella Serovar typhimurium to Trigger Colitis via MyD88-Dependent and MyD88-Independent Mechanisms 1. The Journal of Immunology 174: 1675-1685.

3. Datsenko KA, Wanner BL (2000) One-step inactivation of chromosomal genes in Escherichia coli K-12 using PCR products. Proceedings of the National Academy of Sciences of the United States of America 97: 6640-6645.

Figure S1

Comparison of noise in expression of chromosomal-based and plasmid-based *fliC* promoter.

A. Comparison of noise, as given by coefficient of variation in GFP expression, from the *fliC* promoter on the plasmid pM968 and in the native location on the chromosome of strain M557. Strain M557 (containing no *gfp* gene) and a *rpsM* promoter fused to *gfp+*[28] inserted in the chromosome of strain M557 serve as controls. There is no significant difference in noise between plasmid-based and chromosome-based expression of GFP under the control of the *fliC* promoter. B. Histograms of GFP expression from the *fliC* promoter on the plasmid pM968 (blue lines) and in the native location on the chromosome (green lines). These two strains differ in the average expression level and in the pattern of distribution of the expression levels in the population. Strain M557 containing no *gfp* gene (black line) and a *rpsM* promoter fused to *gfp+* (red line) inserted in the chromosome of strain M557 serve as controls.

Figure S2

Western blot analysis shows that GFP expression correlates with the expression of *FliC*.

Cells containing the *pfliC::gfp* construct in plasmid pM968 were sorted based on expression of GFP using the FACS. Cells were sorted into three fractions, each containing the same number of cells: The first fraction contained cells with high levels of fluorescence; the second fraction contained cells whose fluorescence did not exceed background; the third fraction was a random sample of cells, chosen irrespective of their level of fluorescence. Cells were subjected to western blot analysis with staining using *anti-FliC, -FljB* antibodies and reprobed with *anti*-*OmpC* as a loading control. Only cells with high levels of GFP expression of GFP showed a band when stained with *anti-FliC,* indicating that GFP expression positively correlates with production of *FliC* protein. It is unclear why the fraction containing all cells does not also show a band; however, the lower intensity of the *anti*-*OmpC* band of this fraction and the fact that this fraction contains many cells that do not express gfp suggests that the *anti-FliC* band might be too faint to see.

Figure S3

Lineage tree of microcolony growth and expression pattern of the *dcm* promoter.

GFP expression is plotted in grey (light colored boxes represent high levels of GFP, and dark boxes represent low levels), illustrating the temporal pattern of switching of the *dcm* promoter, isolated from a control population. The image and the lineage tree are based on Movie S2.

Tables

Table S1

Sequenced inserts from selected and control populations and corresponding levels of noise in GFP expression.

Sequence data from the 240 clones used for analysis.

Movies

Movie S1

Time-lapse movie showing GFP expression under the control of the *fliC* promoter during the growth of a microcolony.

GFP is under the control of *fliC* promoter on plasmid M956. This movie lasts for 106 minutes in real time. The phase contrast and fluorescent images have been merged; a lineage reconstruction of this movie can be seen in Fig. 3 in the main text.

Movie S2

Time-lapse movie showing GFP expression under the control of the *dcm* promoter during the growth of a microcolony.

GFP is under the control of the *dcm* (a DNA cytosine methylase) promoter on plasmid M956. This clone was isolated from a control population. This movie lasts for 178 minutes in real time. The phase contrast and fluorescent images have been merged; a lineage reconstruction of this movie can be seen in Figure S3.
